# Novel cerebrospinal fluid inflammatory biomarkers in neonatal post-hemorrhagic hydrocephalus

**DOI:** 10.1186/2045-8118-12-S1-O19

**Published:** 2015-09-18

**Authors:** Gakwaya Habiyaremye, Diego M Morales, Clint D Morgan, James P McAllister, David D Limbrick

**Affiliations:** 1 Department of Neurological Surgery, Division of Pediatric Neurosurgery, Washington University, St. Louis, MO, USA; 2 Vanderbilt University School of Medicine, Nashville, TN, USA

## Introduction

Although inflammation is believed to play a major role in the pathogenesis of post-hemorrhagic hydrocephalus (PHH), a comprehensive characterization of cerebrospinal fluid (CSF) inflammatory biomarkers in PHH has not been performed. Therefore, the aim of this study is to measure concentrations of chemokine and cytokine biomarkers that have yet to be studied in neonatal PHH. These biomarkers include CCL-3, CXCL-12, CX3CL-1, IL-10 and P-selectin.

## Methods

ELISA was used to measure CSF inflammatory biomarker concentrations in 10-15 patients per study group. Study groups included PHH-LP (lumbar puncture) and age-matched pre-term controls (PT). PHH-LP and PT samples were collected perinataly during spinal tap.

## Results

CCL-3, IL-10 and P-selectin were significantly increased in PHH-LP with corresponding p values of 0.0001, 0.0001, and 0.0009. No difference was found in CXCL-12 and CX3CL-1.

## Conclusions

Our findings suggest that CCL-3, IL-10 and P-selectin contribute to the inflammatory process in neonatal PHH. CXCL-12 and CX3CL-1 findings indicate that these biomarkers may not be involved in neonatal PHH inflammatory processes. Interestingly, CCL-3 appears to be an important driver of acute inflammation; it potently attracts neutrophils and other polymorphonuclear-lymphocytes causing secondary injury to brain tissue by generating ROS and secreting pro-inflammatory proteases. These findings suggest that a chronic neuroinflammatory process accompanies PHH and the differential changes in biomarkers help inform future studies of pharmacological modulation.

